# News Items

**Published:** 1917-06

**Authors:** 


### News Items.

### Examination for the Medical Department, U. S. A.

DR. A. F. BEVERLY, MEDICAL EXAMINER FOR AUSTIN DISTRICT.

Men who desire to be examined for the Medical Corps can be examined in Austin by a Federal Examiner, Captain A. F. Beverly, of the Medical Corps. Any reputable physicians beween the age of 21 and 55 are eligible to make application. They will be examined to determine their physical and professional qualifications. Each applicant will be required to fill in a personal history blank and have same sworn before notary public accompanied by certificate from the district clerk of his county showing that he is legally licensed to practice medicine. The application must also be accompanied by letter of recommendation from two citizens in the county showing moral qualifications, sobriety, etc., of the applicant.

The physical examination will be rigid and thorough; professional examination will be oral and of a practical nature. Upon completion of the examination these papers will be forwarded to the Surgeon General at Washington, together with whatever recommendation the examiner will make. The commission will be issued to the applicant in grade no lower than first lieutenant.

The Medical Department of the United States Army is making strong efforts to induce physicians to enter the service. They are especially desirous of securing the services of young men below the ages of 35 for active field service. The Surgeon General's office has been literally swamped by doctors who are desirous of performing some service for their country, but they cannot be used unless they join the Medical Reserve Corps.

It is thought that there will be established in the near future a training camp for medical officers and those who desire to take advantage of this wonderful training should not wait, *because the country needs doctors now.*

Application blanks will be furnished to all physicians, and examinations made without further reference of the Surgeon General's office by the boards of this State, which are located as follows: Austin, A. F. Beverly, Scarbrough Building; Dallas, V. W. Loomis, 236 Page Avenue; Fort Bliss, Commandant Officer, Base Hospital; Fort Crockett, the Surgeon; Fort Sam Houston, the Commandant Officer, Base Hospital.

Those desiring to serve their country should send in their applications at once to the nearest examiner.

---

Kerrville will have an addition to the Kerrville Sanitarium that will triple the present capacity.

---

Dr. Brewer in the *Journal of Hygiene* has suggested as a cure for consumption a diet of peanuts.

---

Texas has been asked by the Federal government to supply at least 300 army doctors and surgeons for immediate service.

---

Mrs. Lucie Castleman, of San Antonio, has offered General Pershing the use of her ranch as a sanitarium for soldiers during the war.

---

The local fishermen of Dallas may be forced to find a substitute for minnows as bait, if a recently instituted health department regulation is enforced, as certain species of these diminutive fish have proven to be excellent mosquito destroyers.

---

At the semi-annual convention of the Texas Surgical Society, war surgery was chiefly discussed by the members.

---

A movement has been started for the securing of 7000 medical men in the Southern department for the great army to be raised in the United States.

---

The Texas Surgical Society during their convention pledged support to President Wilson in the war with Germany and offered the services of the society to the war and navy departments.

---

The announcement has been made that the Texas doctors who have applied for commissions in the Medical Reserve Corps will be sent to the battle fronts of Europe within a comparative short time.

---

The plans for holding a summer session for the purpose of training men for the army and navy has been abandoned by the State Medical College as a suggestion of the Council of National Defense.

---

Major Holman Taylor of the Committee of American Physicians for Preparedness has established headquarters in Fort Worth, where he will receive applications of those who are willing and eligible for service during the war.

---

It is reported by the Rockefeller Institute of Medical Research that wild rats in this country have what is known as Weil's disease—a disease that is prevalent in the European war zone, and it is feared that it will spread to the established camps in this country if an effort is not made to check it.

---

In his appeal for 2000 doctors, Dr. Franklin Martin makes the following statement:

"Six Red Cross base hospital units with twenty-four doctors, fifty nurses and a supporting personnel, aggregating 196 each, have been ordered by the War Department of France for immediate service."

The Bee County Medical Society is ready to serve their country with willingness should the call come.

---

Houston is to have a sanitary training detachment, which will soon see active service, according to a letter received by the Treasurer from C. H. Connor, Major Medical Corps, United States Army, confirming the appointment of Drs. Cody and Fred R. Lummis as commandant of the detachment. The detachment will probably have the same status in the army as a regular field hospital. No definite instructions have been received from headquarters, but it is supposed that when the muster roll of the detachment is completed and it is accepted by the National Red Cross Society it will be transferred either to some training camp or direct to the battlefields of France. The detachment will be composed largely of Houston men.

---

This is a message to all good Americans from the United States government.

That message is, "Buy a Liberty Bond."

It is your duty and your privilege.

It is good Americanism and good business.

Good Americanism because by subscribing you will be doing your "bit" towards making the world "safe for democracy," as our President has so aptly phrased it.

Good business, because the bond bears $3\frac{1}{2}$ per cent interest per annum, and is free from taxation. You can obtain a bond for as small amount as $50 and you do not have to pay for it all at once.

Many a person will keep their bonds and hand them on to their children with pride, as mementos of the world crisis, and as proof that the purchaser did his "bit" for America and for humanity.

Possibly, however, you may need money at some time. Take your bond to the bank and borrow on it. It is good collateral.

Go to your bank tomorrow and file your application. You have to pay only 2 per cent of the amount of your purchase.

Englishmen and Frenchmen are doing their "bit." American citizens, do yours! What you do will be done for our country, our President, for democracy, for humanity.

Fellow citizens, let us do our duty.

### Personals.

Dr. R. L. Ramsdell, of Plainview, has moved with his family to San Augustine, where he will make his home.

Dr. E. J. Britts, of Cisco, has been accepted as a surgeon for the reserve army list.

Dr. L. D. Parnell, of Waxahachie, has enlisted for war service. Dr. Parnell hopes to be sent to the tropics to relieve some of the army surgeons.

Dr. R. A. Largent, of McKinney, has answered the call to the colors and will take the three months officers' training at Leon Springs.

Dr. W. M. Brumby has a new boy born the 23d of April. He has been christened Birto Ralston Brumby.

---

A man rushed to the entrance of a lunatic asylum in the middle of the night and yelled to the keeper to let him in.

'Let me in!" he cried. "I have suddenly gone insane."

The keeper woke up, thrust his head out of a first story window and bellowed down in a rage:

"What? Come here at this time of night? Man, you must be crazy!"—*Wichita Falls Tribune.*

